# Optimizing the Straight Leg Raise Maneuver to Improve Prediction of Conclusive Gastro‐Esophageal Reflux Disease

**DOI:** 10.1111/nmo.70102

**Published:** 2025-06-12

**Authors:** Stefano Siboni, Carlo Galdino Riva, Roberta De Maron, Marco Sozzi, Pierfrancesco Visaggi, Edoardo Savarino, Emanuele Asti, C. Prakash Gyawali

**Affiliations:** ^1^ Division of General and Emergency Surgery, IRCCS Policlinico San Donato, Milano University of Milan Milan Italy; ^2^ Division of Gastroenterology, Department of Traslational Research and New Technologies University of Pisa Pisa Italy; ^3^ Gastroenterology Unit, Department of Surgery, Oncology and Gastroenterology University of Padua Padua Italy; ^4^ Division of Gastroenterology Washington University School of Medicine St. Louis Missouri USA

**Keywords:** esophago‐gastric junction, gastro‐esophageal reflux disease, high‐resolution manometry, reflux monitoring study, straight leg raise maneuver

## Abstract

**Background:**

Gastroesophageal reflux disease (GERD) arises from dysfunction of the anti‐reflux barrier. While high‐resolution manometry (HRM) is routinely performed in suspected GERD, traditional metrics are poor predictors. The routine adoption of the straight leg raise (SLR) maneuver has unveiled a series of unaddressed challenges. This study aimed to determine the minimum intra‐abdominal pressure (IAP) increase for effectively stressing the esophago‐gastric junction (EGJ), the optimal intra‐esophageal pressure (IEP) increase threshold for GERD prediction, and explore EGJ behavior in GERD and non‐GERD patients.

**Methods:**

We performed a retrospective review of HRM tracings and pH‐impedance studies of consecutive adult patients with GERD symptoms. Peak and mean IAP and IEP during the reference period and SLR were recorded. The SLR was effective if the EGJ‐contractile integral (EGJ‐CI) during the maneuver increased by 50% compared to baseline. GERD was diagnosed based on Lyon 2.0. The optimal thresholds for effective and positive SLR were determined using the receiver operator characteristics analysis.

**Results:**

Among the 298 patients included (53% females, age 52 years, BMI 24 kg/m^2^), 145 had GERD. A delta mean IAP increase of 15.4 mmHg best predicted effective EGJ challenge (AUC 0.825). A delta peak IEP increase of 11.4 mmHg optimally predicted GERD (AUC 0.777), aligning with the prior threshold (11 mmHg). Non‐GERD patients exhibited higher EGJ‐CI during SLR, reflecting intact anti‐reflux barrier function.

**Conclusions:**

The SLR maneuver enhances GERD diagnosis in HRM by dynamically assessing EGJ competence. Our study validated the thresholds to define an effective (mean IAP increase of 15.4 mmHg) and positive (peak IEP increase of 11.4 mmHg) maneuver.

## Graphical abstract

The SLR maneuver is a valuable component of GERD evaluation. By establishing optimal thresholds for both effective EGJ challenge and accurate GERD prediction, we provide another step for standardized implementation in clinical practice.

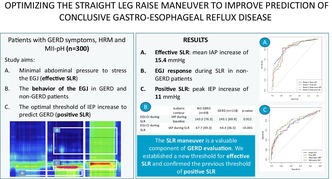

**Summary**

- The straight leg raise should be considered effective if increases the mean abdominal pressure by 15.4 mmHg.
- The straight leg raise should be considered positive if increases the peak esophageal pressure by 11 mmHg.
- The EGJ behave differently in GERD and no‐GERD patients during straight leg raise maneuver.

## 1 Introduction

The anti‐reflux barrier (ARB) is a complex structure that prevents the retrograde movement of gastric contents to the esophagus. This sophisticated mechanism relies on the integrity and synergy of its components, the crural diaphragm (CD) and the lower esophageal sphincter (LES) [1, 2, 3]. Disruption or lack of coordination in these elements represents the primary underlying mechanism of gastroesophageal reflux disease (GERD) [4].

While both the Lyon 2.0 and Porto consensus recommend endoscopic or reflux monitoring criteria for GERD diagnosis, high‐resolution manometry (HRM) has become a routine component of GERD evaluation, particularly before anti‐reflux surgery (ARS) [5, 6]. The primary objectives of HRM include identifying major motility disorders and facilitating precise catheter placement for ambulatory reflux monitoring [7, 8].

Historically, attempts to identify robust HRM metrics for objective GERD diagnosis have been unsuccessful. In 2015, Van Hoeij et al. developed a predictive model incorporating hiatal hernia, LES pressure, and esophageal body amplitude, which demonstrated poor GERD prediction with a significant patient overlap between GERD and non‐GERD patients [9]. A few years later, Masuda et al. published a manometric index that marginally improved previous results, though still failing to achieve adequate diagnostic accuracy [10].

A promising HRM maneuver with the potential to predict GERD emerged with the introduction of the straight leg raise (SLR) maneuver in HRM protocols [11, 12, 13], building upon limited observations from conventional manometry [14, 15]. This maneuver increases intra‐abdominal pressure (IAP) by elevating the legs with the patient in a supine position, thus challenging the esophago‐gastric junction (EGJ) barrier. In case of incompetence of the EGJ barrier, the increased IAP is transmitted backward into the esophagus, resulting in increased intra‐esophageal pressure (IEP). A recent multicenter prospective study demonstrated a strong predictive value of SLR for GERD (sensitivity 79%, specificity 85%), establishing an optimal cut‐off of IEP increase during the maneuver of 11 mmHg [13].

The routine adoption of the SLR underlined a series of unaddressed technical and clinical challenges. Uncertainties persist regarding the minimal abdominal pressure required to effectively stress the EGJ, the differential EGJ behavior in GERD and non‐GERD patients, and the definitive confirmation of the optimal IEP increase threshold for GERD prediction.

Thus, the primary aims of this study were to determine the minimum IAP increase threshold required to effectively challenge the EGJ during the SLR maneuver, and to confirm the optimal IEP increase to predict pathologic GERD as defined by the Lyon Consensus 2.0 [5]. Secondary aims were to explore EGJ behavior during the maneuver and to assess the diagnostic accuracy of biphasic IEP patterns during SLR, when present.

## 2 Methods

Consecutive patients presenting to the tertiary care physiology lab of the IRCCS Policlinico San Donato between December 2021 and December 2024 were retrospectively enrolled. Inclusion criteria consisted of age between 18 and 75 years, HRM and pH‐impedance study (MII‐pH) performed within 2 weeks of each other for persistent GERD symptoms, and the ability to perform the SLR maneuver during HRM. In addition, only HRM and pH‐impedance studies that had not been included in the validation study of the SLR maneuver were eligible for inclusion [13]. Patients with prior foregut surgery, severe obesity (body mass index (BMI) > 35 kg/m^2^), paraesophageal hiatal hernia, scleroderma, eosinophilic esophagitis, and achalasia were excluded. Objective GERD was defined as Los Angeles grades B, C, or D esophagitis, biopsy‐proven Barrett's esophagus, or peptic stricture on endoscopy or acid exposure time (AET) > 6% on MII‐pH monitoring [16]. Moreover, patients with inconclusive AET (4%–6%) and Lyon 2.0 adjunctive criteria (total number of reflux episodes > 80/day, mean nocturnal baseline impedance (MNBI) < 1500 Ω, or positive reflux‐symptom association) were also included in the GERD group. GERD was excluded when AET < 4% [5].

The study protocol was approved by the internal review board and was conducted following the Declaration of Helsinki.

### 2.1 Clinical Evaluation

Clinical and demographic data collected for each patient included age, BMI, waist circumference, symptom onset, smoking habit, primary symptom, upper‐gastrointestinal endoscopy findings (hiatal hernia, Barrett's esophagus, esophagitis), and PPI dose, duration, and effectiveness. Symptoms were assessed using validated questionnaires, including GERD‐Q, GERD Health‐Related Quality of Life (GERD‐HRQL), and Reflux Symptom Index (RSI) [17, 18, 19].

### 2.2 High‐Resolution Esophageal Manometry

HRM was performed with the Medtronic system (Duluth, GA), using a solid‐state catheter with 36 circumferentially incorporated sensors spaced at 1‐cm intervals, and following the standardized protocol defined by the Chicago Classification 4.0 (CCv4.0) [20]. HRM catheters were introduced by experienced physicians after an overnight fast. Ten swallows of 5 mL of room temperature water were performed in the primary position (upright or recumbent), followed by 5 swallows in the secondary position. Each swallow was categorized as intact, weak, or failed based on the distal contractile integral (DCI) (≥ 450 mmHg*cm*sec, 100–450 mmHg*cm*s, and ≤ 100 mmHg*cm*s, respectively). Ineffective esophageal motility (IEM) was defined as more than 70% weak swallows or at least 50% failed swallows. LES characteristics, including total and intra‐abdominal length, basal pressure, EGJ‐contractile integral (EGJ‐CI), and EGJ morphology, were collected. The EGJ‐CI was calculated during quiet rest by including the EGJ in the DCI toolbox for 3 respiratory cycles using the isobaric contour at the gastric pressure and dividing the resultant value by duration [21]. Multiple rapid swallows, consisting of 5 swallows of 2 mL of water administered 2–3 s apart, were also performed. Contraction reserve was defined as a ratio between MRS‐DCI and the mean ten swallows DCI > 1.

### 2.3 Straight Leg Raise Maneuver

Upon completion of the standard Chicago Classification 4.0 protocol, patients were asked to perform the SLR maneuver as previously described [13]. With the patient in the supine position, both legs were raised at an angle of 45° for at least 5 s. The maneuver was repeated after 30 s, and the first adequate maneuver was included in the analysis. IEP and IAP were analyzed both at baseline and during SLR. IEP was measured as peak and mean pressure over 5 s, 5 cm above the proximal margin of the LES. IAP was measured as peak and mean over 5 s, 1 cm below the distal margin of the CD impression. All the HRM plots were analyzed *de novo* for the purpose of the study by expert physicians (SS and MS).

### 2.4 Esophageal pH and pH‐Impedance Study

All patients were asked to discontinue acid‐suppressive drugs for at least 2 weeks before reflux monitoring studies [6, 22]. Rescue therapy protocol included the use of alginate‐based medications [23]. The MII‐pH studies were performed using catheters with 8 impedance and 2 pH electrodes. The catheter was calibrated using buffer solutions at pH of 4.0 and 7.0 and then inserted transnasally with the esophageal pH electrode 5 cm above the proximal margin of the LES. The patients were asked to avoid acidic food and drinks and to record symptoms, duration of meal, and time spent in the recumbent position either in a diary or on the recorder itself.

Total, upright, and recumbent AET, DeMeester score, number of acid, weakly acid, weakly alkaline reflux episodes, and reflux symptom association were collected. Reflux‐symptom association was defined as positive symptom index (SI) or symptom association probability (SAP).

Data were analyzed with a dedicated software, using Wingate consensus criteria [24]. Additionally, the MNBI and the post‐reflux swallow‐induced peristaltic wave (PSPW) index were recorded [25, 26].

### 2.5 Data Collection and Statistical Analysis

Categorical variables are presented as frequency and percentages while continuous variables as median and interquartile range (IQR). Categorical variables were compared using the chi‐squared test or the Fisher exact test as appropriate. The normality of continuous variables was assessed with the Shapiro–Wilk test. Normal variables were compared with the *t*‐test and nonparametric variables using the Kruskal–Wallis test.

The study population was stratified according to GERD diagnosis. Demographic and clinical characteristics were compared. The performances of various criteria of effective SLR were compared based on their ability to challenge the EGJ using receiver operator characteristics (ROC) analysis. The EGJ was considered adequately challenged when the EGJ‐CI increased by at least 50% compared to baseline during the SLR maneuver. The rationale for using this criterion was the observation that in all the patients with an actual IAP increase during SLR, we noticed an augmented high‐pressure zone. The augmentation of the EGJ‐CI during the maneuver is a physiologic effect of the increased IAP that occurs regardless of EGJ integrity.

The influence of BMI, waist circumference, and age on mean IAP increase was evaluated.

To identify the optimal metric for predicting objective GERD, all the possible relationships between peak and mean IEP and IAP during SLR and baseline were investigated in patients with effective SLR. The optimal criteria and threshold were determined through ROC analysis, using Youden's index. Bonferroni correction for multiple comparisons has been applied when appropriate.

To assess the independent contribution of SLR maneuver to GERD diagnosis, a logistic regression model has been developed.

**FIGURE 1:**
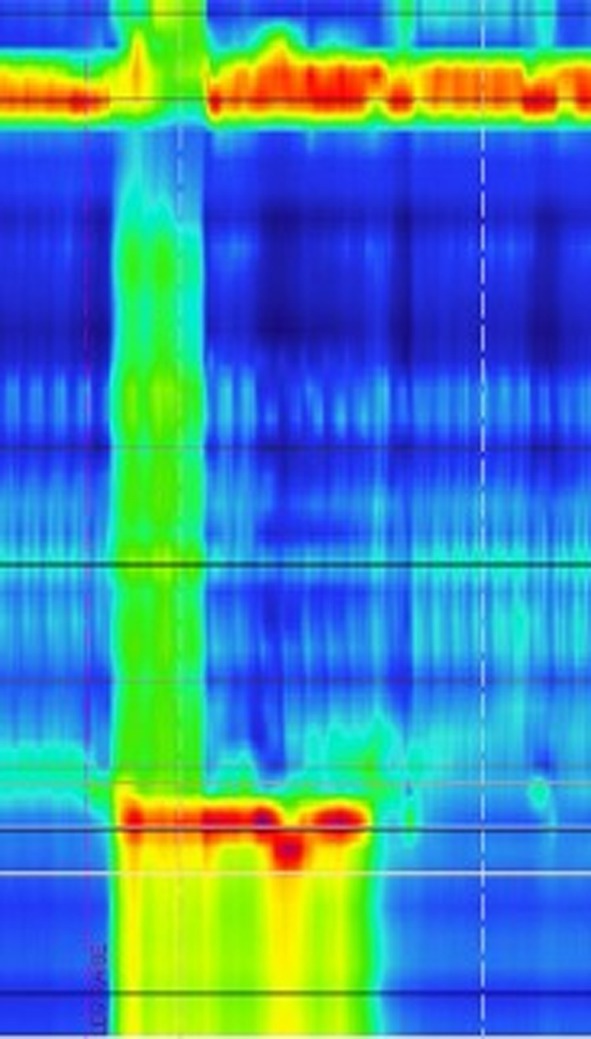
Biphasic IEP pressure during SLR.

To evaluate differential EGJ behavior in GERD and non‐GERD patients, the EGJ‐CI was calculated utilizing gastric pressure as the isobaric contour during baseline and SLR. In patients with a biphasic IEP pressure during SLR (initial higher and a final lower IEP), the diagnostic ability of the two patterns was evaluated (Figure 1).

All statistical tests were 2‐sided. *p* < 0.05 was considered statistically significant. All analyses were performed using R software version 3.6.1 (The R Foundation for Statistical Computing, Vienna, Austria).

## 3 Results

Over the study period, 298 patients (53% females, median age 52 years, median BMI 24 kg/m^2^) were enrolled. Patients with pathologic GERD (145 patients) were more frequently males (56% vs. 38%, *p* = 0.002), older (54 vs. 48 years, *p* = 0.006), with higher BMI (25.3 vs. 22.8 kg/m^2^, *p* < 0.001) and larger waist circumference (96 vs. 85 cm, *p* < 0.001) compared to non‐GERD (Table 1). All GERD‐related HRM variables and MII‐pH variables evaluated were significantly different between the two groups (Table 2).

**TABLE 1:** Demographic and endoscopic characteristics of the study population. Continuous values are expressed as median [IQR].

	Total (*n* = 298)	No GERD (*n* = 153)	GERD (*n* = 145)	*p*
Male, *n* (%)	139 (47)	58 (38)	81 (56)	0.002
Age, (years)	52 [24]	48 [27]	54 [20]	0.006
BMI, (Kg/m^2^)	24.0 [5.6]	22.8 [4.6]	25.3 [5.2]	< 0.001
Waist circumference, (cm)	93 [15]	85 [17]	96 [12]	< 0.001
Symptoms duration, (months)	24 [52]	24 [55]	36 [73]	0.031
PPI use, *n* (%)	244 (86)	126 (86)	118 (87)	0.8
Response to PPI				0.13
No response, *n* (%)	77 (33)	43 (36)	34 (30)	
Partial response, *n* (%)	84 (36)	47 (39)	37 (33)	
Full benefit, *n* (%)	72 (31)	30 (25)	42 (37)	
Endoscopic findings				
Hiatal hernia, *n* (%)	130 (48)	50 (36)	80 (60)	< 0.001
Esophagitis, *n* (%)	69 (23.2)	20 (13.1)	49 (33.8)	< 0.001
Grade A	43 (62.3)	20 (100)	23 (47)	
Grade B	20 (28.9)	0 (0)	20 (40.7)	
Grade C	4 (5.9)	0 (0)	4 (8.2)	
Grade D	2 (2.9)	0 (0)	2 (4.1)	
Questionnaires				
Gerd‐Q	8.0 [5.0]	7.0 [4.3]	8.0 [5.8]	0.040
GERD‐HRQL	13 [16]	12 [15]	14 [16]	0.11
RSI	8 [13]	9 [14]	7 [12]	0.4
GSS	60 [50]	50 [55]	70 [50]	0.011

Abbreviations: BMI, body mass index; GERD, gastro‐esophageal reflux disease; GERD‐HRQL, GERD health‐related quality of life; GSS, global symptom severity; PPI, proton pump inhibitors; RSI, Reflux symptom index.

**TABLE 2:** HRM and pH‐impedance characteristics of the study population. Continuous values are expressed as median [IQR].

HRM and pH data	Total (*n* = 298)	No GERD (*n* = 153)	GERD (*n* = 145)	*p*
HRM findings				
EGJ type				< 0.001
1, *n* (%)	169 (57)	110 (72)	59 (41)	
2, *n* (%)	90 (30)	42 (27)	48 (33)	
3, *n* (%)	39 (13)	1 (0.7)	38 (26)	
Hiatal hernia, *n* (%)	136 (46%)	48 (31%)	88 (61%)	
Hiatal hernia size, (cm)	0.30 [1.45]	0.00 [0.58]	1.20 [1.90]	< 0.001
LES total length, (cm)	1.80 [0.68]	1.90 [0.70]	1.70 [0.40]	< 0.001
LES intra‐abdominal length, (cm)	0.10 [1.20]	0.70 [1.50]	0.00 [0.60]	< 0.001
LES basal pressure, (mmHg)	22 [18]	27 [19]	17 [16]	< 0.001
EGJ‐CI, (mmHg*cm)	31 [34]	37 [40]	26 [25]	< 0.001
Patients with IEM, *n* (%)	67 (22)	22 (14)	45 (31)	< 0.001
pH‐impedance findings				
Acid exposure time, (%)	6 [10]	2 [3]	12 [10]	< 0.001
AET > 6%	139 (47)	0 (0)	139 (96)	< 0.001
Total reflux episodes, *n*	31 [29]	23 [24]	38 [29]	< 0.001
Acid reflux episodes, *n*	23 [26]	14 [18]	32 [25]	< 0.001
MNBI, (Ω)	2320 [2455]	3510 [1690]	1170 [1285]	< 0.001
MNBI < 1500 Ω, *n* (%)	96 (34)	8 (5.6)	88 (63)	< 0.001
Reflux‐symptom association, *n* (%)	31 (20)	6 (8.2)	25 (30)	< 0.001
Total reflux episodes > 80, *n* (%)	6 (2.3)	0 (0)	6 (4.7)	0.014
DeMeester score	21 [33]	9 [10]	44 [35]	< 0.001

Abbreviations: AET, Acid exposure time; EGJ, Esophago‐gastric junction; EGJ‐CI, EGJ‐contractile integral; GERD, Gastro‐esophageal reflux disease; HRM, High‐resolution manometry; IEM, Ineffective esophageal motility; MNBI, Mean nocturnal baseline impedance.

**FIGURE 2:**
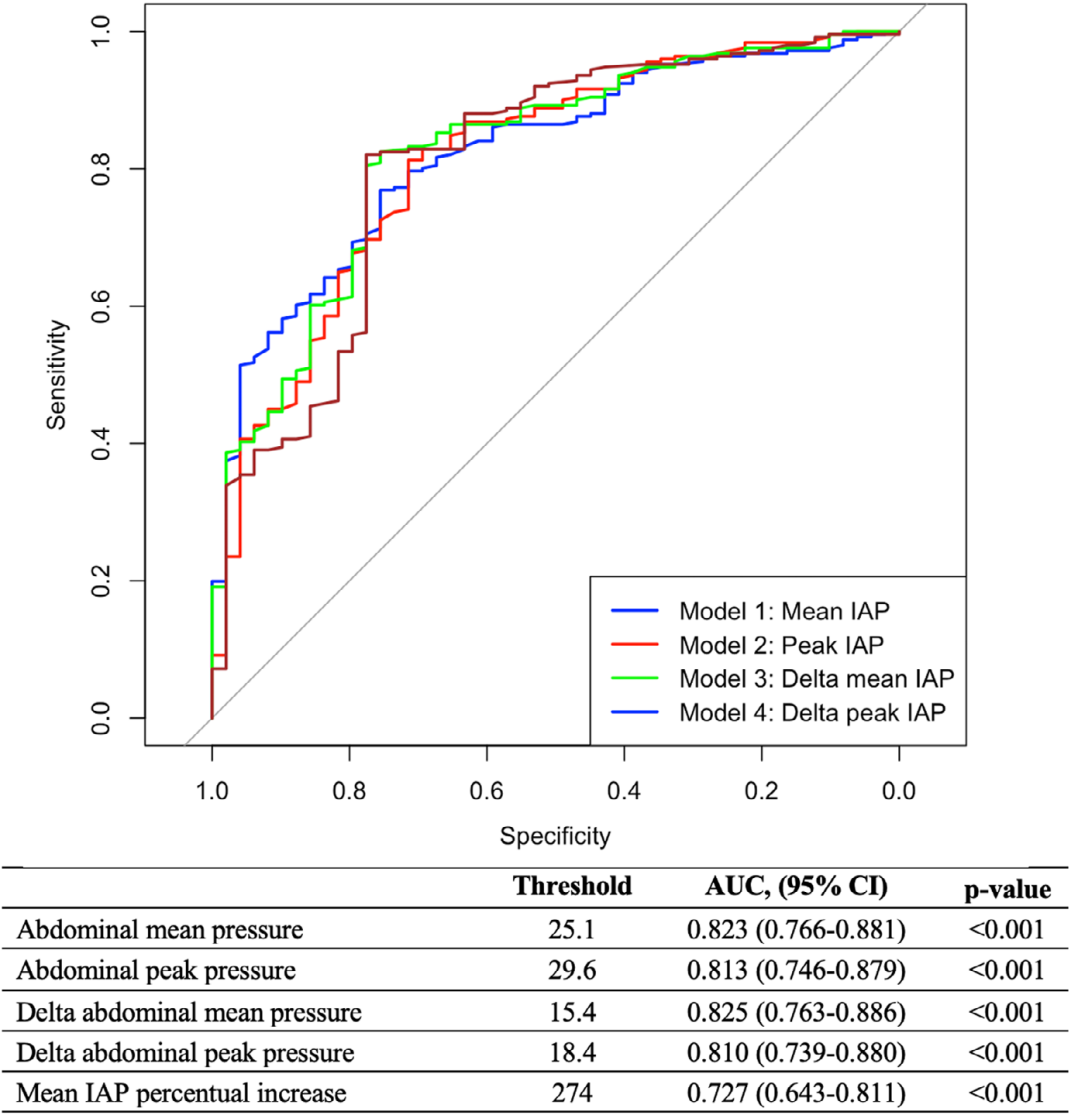
ROC analysis testing different measures of abdominal pressure increase to effectively stress the esophago‐gastric junction.

The best predictor of effective SLR was delta mean IAP increase (mean IAP during SLR—mean IAP at baseline) (AUC 0.825, CI 0.763–0.886, optimal threshold of 15.4 mmHg, sensitivity 80.5%, specificity 77.6%) (Figure 2). With increasing IAP thresholds, the SLR demonstrated greater accuracy in predicting pathologic GERD, though the number of patients with an effective SLR decreased (Table 3). Mean IAP increase was influenced by BMI (*r* = 0.21, *p* < 0.001) and waist circumference (*r* = 0.25, *p* = 0.003) but not by age (*r* = 0.06, *p* = 0.311) (Figure S1).

**TABLE 3:** ROC analysis testing the ability of the SLR to predict pathologic GERD in different subsets of patients, selected according to different IAP increase criteria and thresholds.

	Patients	AUC (95% CI)	*p*
Mean IAP increase > 50% (current definition)	296 (98.7)	0.738 (0.681–0.796)	< 0.001
Mean IAP increase > 100%	265 (88.3)	0.734 (0.673–0.795)	< 0.001
Mean IAP increase > 200%	213 (71.0)	0.789 (0.727–0.852)	< 0.001
Mean IAP during SLR 20 mmHg	236 (78.7)	0.770 (0.709–0.831)	< 0.001
Mean IAP during SLR 30 mmHg	163 (54.3)	0.799 (0.731–0.868)	< 0.001
Mean IAP during SLR 40 mmHg	83 (27.7)	0.878 (0.803–0.953)	< 0.001
IAP during SLR increase of 10 mmHg	251 (83.7)	0.766 (0.707–0.825)	< 0.001
IAP during SLR increase of 15.4 mmHg	217 (72.3)	0.825 (0.763–0.886)	< 0.001
IAP during SLR increase of 20 mmHg	183 (61)	0.837 (0.777–0.869)	< 0.001
IAP during SLR increase of 30 mmHg	98 (32.7)	0.867 (0.796–0.938)	< 0.001

**FIGURE 3:**
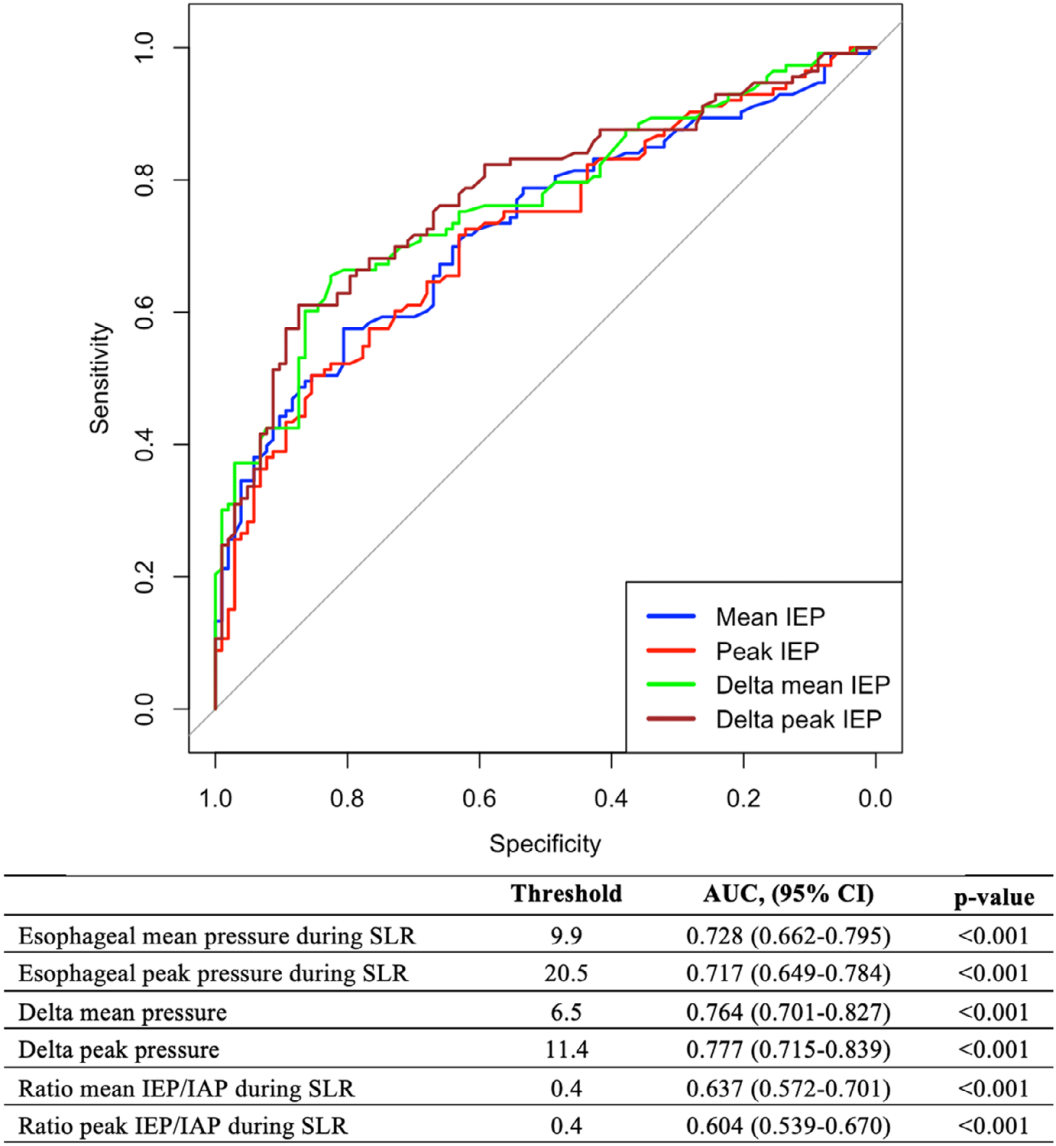
ROC analysis testing different measures of esophageal pressure to predict pathologic GERD.

In the 217 patients with an effective SLR, all possible relationships between peak and mean IEP and IAP during SLR and at baseline segregated GERD patients from non‐GERD (Table 4). Among these, delta peak IEP increase (peak IEP during SLR—peak IEP during baseline) was the best predictor of pathologic GERD (AUC 0.777, CI 0.715–0.839, optimal threshold of 11.4 mmHg, sensitivity 56.7%, specificity 86.8%) (Figure 3). After Bonferroni correction for multiple comparisons, the delta peak IEP remained statistically significant (*p* < 0.001).

**TABLE 4:** Esophageal pressure measurements in patients with effective SLR (delta IAP 15.4 mmHg).

	NO GERD (*n* = 99)	GERD (*n* = 118)	*p*
Esophageal mean pressure during SLR	4.2 [7.8]	12.7 [20.0]	< 0.001
Esophageal peak pressure during SLR	11 [11.0]	20 [23.8]	< 0.001
Delta mean pressure	1.3 [5.8]	9.3 [21.6]	< 0.001
Delta peak pressure	3 [8.1]	14 [20.3]	< 0.001
IEP/IAP (mean)	0.33 [0.38]	0.44 [0.50]	< 0.001
IEP/IAP (peak)	0.15 [0.29]	0.32 [0.51]	< 0.001

**TABLE 5:** Logistic regression including demographic and manometric variables associated with GERD.

Variable	Odds ratio	95% CI	*p*
Age	1.01	0.94–1.07	0.907
BMI	1.33	0.97–1.93	0.088
Waist circumference	1.05	0.94–1.96	0.412
Positive SLR	6.45	1.25–12.46	0.038
Manometric Hiatal hernia	4.45	1.20–8.95	0.044

Abbreviations: BMI, Body mass index; SLR, Straight leg raise maneuver.

On the multivariate model, a positive SLR maneuver and hiatal hernia were independent predictors of pathologic GERD (OR: 6.45 and 4.45, respectively) (Table 5).

On analysis of EGJ contractility, baseline EGJ‐CI above basal‐IAP was lower in the GERD group (*p* = 0.912). Similarly, the SLR‐EGJ‐CI calculated above SLR‐IAP was also lower in the GERD group (< 0.001) (Table 6).

**TABLE 6:** Contractile indices (EGJ‐CI at different isobaric contours) in patients with effective SLR (delta IAP 15.4 mmHg).

	Isobaric contour	NO GERD (*n* = 99)	GERD (*n* = 118)	*p*
EGJ‐CI during baseline	IAP during baseline	34 [38]	26 [26]	< 0.001
EGJ‐CI during SLR	IAP during baseline	143.5 [76.3]	143.1 [69.9]	0.912
EGJ‐CI during SLR	IAP during SLR	67.7 [49.3]	44.4 [36.5]	< 0.001

In 31 patients (10%), a double IEP pattern was demonstrated during SLR, with an initial higher and a final lower IEP. The diagnostic prediction of GERD of the two phases of SLR using AUC on ROCs were 0.610 and 0.777, respectively.

## 4 Discussion

In this retrospective observational study, we demonstrate the minimum IAP increase threshold to effectively challenge the EGJ during the SLR maneuver; we confirm the previously established threshold of a positive SLR maneuver in an independent cohort [13] and enhance the evaluation of EGJ behavior in GERD and non‐GERD patients, endorsing the SLR maneuver as a valuable component of HRM protocols for GERD evaluation.

Interestingly, the best criterion (delta peak IEP increase during SLR) and threshold (11.4 mmHg) were similar to those found in the previous study (optimal cut‐off 11 mmHg), demonstrating strong predictive value in distinguishing GERD from non‐GERD patients [13]. Given the multicenter nature of the SLR international study and the very similar value found in the present study, we believe that the threshold of 11 mmHg should not be modified [13].

Among patients with effective SLR, the ability to predict pathologic GERD was marginally inferior to the original paper (AUC 0.777 vs. 0.840), potentially related to a slightly different population enrolled for the current study. However, the GERD prediction ability of this simple maneuver remains significantly higher than previously proposed comprehensive indices of HRM parameters [9, 10].

The introduction of SLR represents a significant advancement over previous attempts, which have historically demonstrated limited clinical utility. Earlier predictive models incorporating parameters such as hiatal hernia, LES pressure, and esophageal body amplitude showed poor diagnostic accuracy with substantial patient overlap between GERD and non‐GERD populations. Both the indexes proposed by Van Hoeij et al. and Masuda et al. failed to achieve adequate sensitivity and specificity for clinical application, mainly because they rely on static parameters (LES pressure, body contractility, static thoraco‐abdominal gradient) [9, 10]. Our study's success in achieving higher AUC values can be attributed to the SLR's ability to dynamically challenge the EGJ, revealing pressure gradients that static measurements fail to capture. This dynamic approach leverages the physiological principle that GERD results from a failure of the ARB under stress [15], a concept supported by the significant differences in EGJ contractility (EGJ‐CI) observed between GERD and non‐GERD patients during SLR.

Our analysis of EGJ behavior during SLR revealed distinct patterns in GERD and non‐GERD patients, with both groups exhibiting similar EGJ‐CI values when calculated above baseline IAP. However, when using SLR‐induced IAP as the reference, non‐GERD patients demonstrated significantly higher EGJ‐CI values, reflecting an intact ARB capable of withstanding increased IAP. This finding elucidates the underlying pathophysiological mechanism distinguishing GERD from non‐GERD patients—not merely EGJ weakness at baseline, but specifically impaired contractile augmentation in response to increased IAP. This concept aligns with historical physiologic studies that demonstrated an activation of the CD during SLR in healthy subjects [27].

One of the main limitations of SLR that limited its common use in clinical practice was the definition of the effectiveness of the maneuver. Based on expert opinion, until now, the definition of effectiveness has been an increase of 50% of IAP during the maneuver compared to baseline [11]. However, in the case of low mean IAP during baseline, a very small increment would fit the definition without effectively challenging the EGJ. Our study showed that the best criterion to effectively challenge the EGJ is the delta mean IAP increase (AUC 0.825), with an optimal threshold of 15.4 mmHg. This threshold optimization has the potential to standardize the use of the SLR maneuver in clinical settings.

The gradient of accuracy in predicting pathologic GERD observed with increasing IAP thresholds deserves particular attention (Table 3). While the highest IAP increase tested (mean IAP of 40 mmHg) yielded superior GERD predictive accuracy, this was achieved in only 27.7% of patients. Conversely, a 15.4 mmHg IAP increase threshold balanced diagnostic accuracy with clinical feasibility and was observed in 72.3% of patients. This practical consideration is essential for widespread clinical implementation but, at the same time, it highlights the notion that the higher the abdominal pressure, the higher the predictive ability of the maneuver. This finding emphasizes the importance of increasing IAP as much as possible, supporting the use of double leg raise (i.e., elevating both legs at the same time) to maximally augment IAP [28].

The logistic regression analysis indicates that a positive SLR maneuver (OR: 6.45) remains an independent predictive factor of objective GERD in a model corrected for BMI, age, waist circumference, and, most importantly, manometric hiatal hernia. This underlines the SLR's ability to assess EGJ impairment, regardless of its anatomical integrity. Consistently, in a recent paper, Ferrari et al. showed the ability of the Milan Score, a manometric comprehensive parameter primarily driven by the SLR maneuver, to effectively distinguish GERD in patients with EGJ type II morphology [29, 30].

Our study identified a small but interesting subset of patients (10%) exhibiting biphasic IEP patterns during SLR, with an initial higher peak followed by lower pressures. This group showed markedly reduced diagnostic accuracy if the IEP was calculated during the first part of the maneuver. We speculate that this artifact may be due to excessive chest straining during the initial phase of the maneuver, which naturally decreases with maintenance of the effort.

Our study has important clinical implications, particularly in the context of ARS planning. HRM with the SLR maneuver can identify EGJ incompetence more objectively, guiding surgical decision‐making and helping surgeons to perform a better selection, thus increasing clinical outcomes after surgery. Moreover, the maneuver's simplicity—requiring no additional equipment beyond standard HRM systems—enhances its feasibility for widespread adoption in motility laboratories. Finally, standardization of the SLR maneuver will positively impact the effectiveness and clinical utility of the Milan Score, a comprehensive HRM metric that quantifies ARB disruption and relies heavily on SLR [29].

The strengths of the study include the use of a standardized protocol in a sizable independent cohort not used in the initial SLR validation study [13], analyzed according to international standards (CCv4.0 and Lyon 2.0). In addition, IAP and IEP were collected at multiple points in the HRM plot to identify the best thresholds of effectiveness and positivity of the maneuver.

There are limitations that temper the strength of this study. Despite robust patient numbers and the review of all the HRM studies by study investigators, this was a retrospective single‐center study that will benefit from external validation. Additionally, while we established optimal thresholds for effective SLR and GERD prediction, the standardization of patient positioning, leg elevation angle, and duration remain necessary for consistent clinical application. Finally, the AUC values, while significant, indicate that the maneuver itself is not a perfect predictor of GERD and suggest the need for integration with other HRM parameters (EGJ morphology, IEM, EGJ‐CI), clinical characteristics (age, gender, BMI), and other diagnostic parameters (reflux monitoring study with AET, MNBI, reflux‐symptom association or DeMeester score) to enhance diagnostic accuracy.

In conclusion, this study advances our understanding of the SLR maneuver as a valuable component of comprehensive GERD evaluation. By establishing optimal thresholds for both effective EGJ challenge and accurate GERD prediction, we provide another step for standardized implementation in clinical practice. The ability of the SLR to identify functional EGJ deficiencies not evident during static assessment represents a significant advancement in objective GERD diagnosis, potentially improving patient selection for anti‐reflux interventions and enabling more personalized therapeutic approaches.

## Author Contributions

**Stefano Siboni:** conception of the study, acquisition of the data, analysis and interpretation of the data, drafting the paper, approving the final manuscript. **Carlo Galdino Riva:** acquisition of the data, analysis and interpretation of the data, drafting the paper, approving the final manuscript. **Roberta De Maron:** acquisition of the data, approving the final manuscript. **Marco Sozzi:** drafting the paper, approving the final manuscript. **Pierfrancesco Visaggi:** drafting the paper, approving the final manuscript. **Edoardo Savarino:** drafting the paper, approving the final manuscript. **Emanuele Asti:** drafting the paper, approving the final manuscript. **C. Prakash Gyawali:** conception of the study, drafting the paper, approving the final manuscript.

## Conflicts of Interest

Edoardo Vincenzo Savarino has served as speaker for Abbvie, Abivax, Agave, AGPharma, Alfasigma, Apoteca, Biosline, CaDiGroup, Celltrion, Dr. Falk, EG Stada Group, Fenix Pharma, Galapagos, Johnson & Johnson, JB Pharmaceuticals, Innovamedica/Adacyte, Eli Lilly, Malesci, Mayoly Biohealth, Montefarco, Novartis, Omega Pharma, Pfizer, Rafa, Reckitt Benckiser, Sandoz, Sanofi/Regeneron, SILA, Sofar, Takeda, Tillots, Unifarco; has served as consultant for Abbvie, Agave, Alfasigma, Biogen, Bristol‐Myers Squibb, Celltrion, Dr. Falk, Eli Lilly, Fenix Pharma, Ferring, Giuliani, Grunenthal, Johnson & Johnson, JB Pharmaceuticals, Merck & Co, Nestlè, Pfizer, Reckitt Benckiser, Sanofi/Regeneron, SILA, Sofar, Takeda, Unifarco; he received research support from Bonollo, Difass, Pfizer, Reckitt Benckiser, Sanofi/Regeneron, SILA, Sofar, Unifarco, Zeta Farmaceutici; C.P.G. has consulted for Medtronic, Phathom, Braintree and is a speaker for Carnot.

## Supporting information


**Figure S1.** Relationship between mean intra‐abdominal pressure increase and body mass index (BMI), waist circumference and age.

## Data Availability

The data that support the findings of this study are available on request from the corresponding author. The data are not publicly available due to privacy or ethical restrictions.

## References

[1] D. Stefanidis , W. W. Hope , G. P. Kohn , P. R. Reardon , W. S. Richardson , and R. D. Fanelli , “Guidelines for Surgical Treatment of Gastroesophageal Reflux Disease,” Surgical Endoscopy 24, no. 11 (2010): 2647–2669. 20725747 10.1007/s00464-010-1267-8

[2] N. T. Nguyen , N. C. Thosani , M. I. Canto , et al., “The American Foregut Society White Paper on the Endoscopic Classification of Esophagogastric Junction Integrity,” Foregut: The Journal of the American Foregut Society 2, no. 4 (2022): 339–348, 10.1177/26345161221126961.

[3] R. K. Mittal , D. F. Rochester , and R. W. McCallum , “Sphincteric Action of the Diaphragm During a Relaxed Lower Esophageal Sphincter in Humans,” American Journal of Physiology 256, no. 1 Pt 1 (1989): G139–G144, 10.1152/ajpgi.1989.256.1.G139. 2912145

[4] J. Tack and J. E. Pandolfino , “Pathophysiology of Gastroesophageal Reflux Disease,” Gastroenterology 154, no. 2 (2018): 277–288, 10.1053/j.gastro.2017.09.047. 29037470

[5] C. P. Gyawali , R. Yadlapati , R. Fass , et al., “Updates to the Modern Diagnosis of GERD: Lyon Consensus 2.0,” Gut 73, no. 2 (2024): 361–371, 10.1136/gutjnl-2023-330616. 37734911 PMC10846564

[6] S. Roman , C. P. Gyawali , E. Savarino , et al., “Ambulatory Reflux Monitoring for Diagnosis of Gastro‐Esophageal Reflux Disease: Update of the Porto Consensus and Recommendations From an International Consensus Group,” Neurogastroenterology and Motility 29, no. 10 (2017): 1–15, 10.1111/nmo.13067. 28370768

[7] C. P. Gyawali , S. Roman , A. J. Bredenoord , et al., “Classification of Esophageal Motor Findings in Gastro‐Esophageal Reflux Disease: Conclusions From an International Consensus Group,” Neurogastroenterology and Motility 29, no. 12 (2017): 13104, 10.1111/nmo.13104. 28544357

[8] E. Savarino , N. de Bortoli , M. Bellini , et al., “Practice Guidelines on the Use of Esophageal Manometry – A GISMAD‐SIGE‐AIGO Medical Position Statement,” Digestive and Liver Disease 48, no. 10 (2016): 1124–1135, 10.1016/j.dld.2016.06.021. 27443492

[9] F. B. Van Hoeij , A. J. Smout , and A. J. Bredenoord , “Predictive Value of Routine Esophageal High‐Resolution Manometry for Gastro‐Esophageal Reflux Disease,” Neurogastroenterology and Motility 27, no. 7 (2015): 963–970, 10.1111/nmo.12570. 25930019

[10] T. Masuda , S. K. Mittal , B. Kovacs , et al., “Simple Manometric Index for Comprehensive Esophagogastric Junction Barrier Competency Against Gastroesophageal Reflux,” Journal of the American College of Surgeons 230, no. 5 (2020): 744–755, 10.1016/j.jamcollsurg.2020.01.034. 32142925

[11] B. D. Rogers , A. Rengarajan , I. A. Ali , S. L. Hasak , V. Hansalia , and C. P. Gyawali , “Straight Leg Raise Metrics on High‐Resolution Manometry Associate With Esophageal Reflux Burden,” Neurogastroenterology and Motility 32, no. 12 (2020): e13929, 10.1111/nmo.13929. 32633016

[12] B. Rogers , S. Hasak , V. Hansalia , and C. P. Gyawali , “Trans‐Esophagogastric Junction Pressure Gradients During Straight Leg Raise Maneuver on High‐Resolution Manometry Associate With Large Hiatus Hernias,” Neurogastroenterology and Motility 32, no. 7 (2020): e13836, 10.1111/nmo.13836. 32163648

[13] S. Siboni , I. Kristo , B. D. Rogers , et al., “Improving the Diagnostic Yield of High‐Resolution Esophageal Manometry for GERD: The “Straight Leg‐Raise” International Study,” Clinical Gastroenterology and Hepatology 21, no. 7 (2023): 1761–1770, 10.1016/j.cgh.2022.10.008. 36270615

[14] D. G. Butterfield , J. E. Struthers, Jr. , and J. P. Showalter , “A Test of Gastroesophageal Sphincter Competence. The Common Cavity Test,” American Journal of Digestive Diseases 17, no. 5 (1972): 415–421, 10.1007/BF02231293. 5024985

[15] S. Siboni , L. Bonavina , B. D. Rogers , et al., “Effect of Increased Intra‐Abdominal Pressure on the Esophagogastric Junction: A Systematic Review,” Journal of Clinical Gastroenterology 56, no. 10 (2022): 821–830, 10.1097/MCG.0000000000001756. 36084164 PMC9553247

[16] P. Visaggi , G. Del Corso , C. P. Gyawali , et al., “Ambulatory pH‐Impedance Findings Confirm That Grade B Esophagitis Provides Objective Diagnosis of Gastroesophageal Reflux Disease,” American Journal of Gastroenterology 118, no. 5 (2023): 794–801, 10.14309/ajg.0000000000002173. 36633477

[17] R. Jones , O. Junghard , J. Dent , et al., “Development of the GerdQ, a Tool for the Diagnosis and Management of Gastro‐Oesophageal Reflux Disease in Primary Care,” Alimentary Pharmacology & Therapeutics 30, no. 10 (2009): 1030, 10.1111/j.1365-2036.2009.04142.x. 19737151

[18] V. Velanovich , “The Development of the GERD‐HRQL Symptom Severity Instrument,” Diseases of the Esophagus 20, no. 2 (2007): 130–134, 10.1111/j.1442-2050.2007.00658.x. 17439596

[19] P. C. Belafsky , G. N. Postma , and J. A. Koufman , “Validity and Reliability of the Reflux Symptom Index (RSI),” Journal of Voice 16, no. 2 (2002): 274–277, 10.1016/s0892-1997(02)00097-8. 12150380

[20] R. Yadlapati , P. J. Kahrilas , M. R. Fox , et al., “Esophageal Motility Disorders on High‐Resolution Manometry: Chicago Classification Version 4.0,” Neurogastroenterology and Motility 33, no. 1 (2021): e14058, 10.1111/nmo.14058. 33373111 PMC8034247

[21] F. Nicodème , M. Pipa‐Muniz , K. Khanna , P. J. Kahrilas , and J. E. Pandolfino , “Quantifying Esophagogastric Junction Contractility With a Novel HRM Topographic Metric, the EGJ‐Contractile Integral: Normative Values and Preliminary Evaluation in PPI Non‐Responders,” Neurogastroenterology and Motility 26, no. 3 (2014): 353–360, 10.1111/nmo.12267. 24460814 PMC4605557

[22] E. Savarino , M. Frazzoni , E. Marabotto , et al., “A SIGE‐SINGEM‐AIGO Technical Review on the Clinical Use of Esophageal Reflux Monitoring,” Digestive and Liver Disease 52, no. 9 (2020): 966–980, 10.1016/j.dld.2020.04.031. 32513632

[23] E. Savarino , N. de Bortoli , P. Zentilin , et al., “Alginate Controls Heartburn in Patients With Erosive and Nonerosive Reflux Disease,” World Journal of Gastroenterology 18, no. 32 (2012): 4371–4378, 10.3748/wjg.v18.i32.4371. 22969201 PMC3436053

[24] C. P. Gyawali , B. Rogers , M. Frazzoni , et al., “Inter‐Reviewer Variability in Interpretation of pH‐Impedance Studies: The Wingate Consensus,” Clinical Gastroenterology and Hepatology 19, no. 9 (2021): 1976–1978, 10.1016/j.cgh.2020.09.002. 32890752

[25] M. Frazzoni , E. Savarino , N. de Bortoli , et al., “Analyses of the Post‐Reflux Swallow‐Induced Peristaltic Wave Index and Nocturnal Baseline Impedance Parameters Increase the Diagnostic Yield of Impedance‐pH Monitoring of Patients With Reflux Disease,” Clinical Gastroenterology and Hepatology 14, no. 1 (2016): 40–46, 10.1016/j.cgh.2015.06.026. 26122764

[26] M. Frazzoni , N. de Bortoli , L. Frazzoni , S. Tolone , V. Savarino , and E. Savarino , “Impedance‐pH Monitoring for Diagnosis of Reflux Disease: New Perspectives,” Digestive Diseases and Sciences 62, no. 8 (2017): 1881–1889, 10.1007/s10620-017-4625-8. 28550489

[27] R. K. Mittal , M. Fisher , R. W. McCallum , D. F. Rochester , J. Dent , and J. Sluss , “Human Lower Esophageal Sphincter Pressure Response to Increased Intra‐Abdominal Pressure,” American Journal of Physiology 258, no. 4 Pt 1 (1990): G624–G630, 10.1152/ajpgi.1990.258.4.G624. 2333975

[28] D. S. Rim and H. P. Parkman , “Comparative Evaluation of Single Versus Double Leg Raise Maneuver in High‐Resolution Esophageal Manometry,” Neurogastroenterology and Motility 36, no. 10 (2024): e14868, 10.1111/nmo.14868. 39051711

[29] S. Siboni , M. Sozzi , I. Kristo , et al., “The Milan Score: A Novel Manometric Tool for a More Efficient Diagnosis of Gastro‐Esophageal Reflux Disease,” United European Gastroenterology Journal 12 (2024): 552–561, 10.1002/ueg2.12565. 38536701 PMC11176912

[30] D. Ferrari , S. Siboni , M. Sozzi , et al., “The Milan Score Predicts Objective Gastroesophageal Reflux Disease in Patients With Type 2 Esophagogastric Junction,” Neurogastroenterology and Motility (2025): e14987, 10.1111/nmo.14987. 39757994 PMC12849986

