# Supplementary figures and images for: Optimizing the Straight Leg Raise Maneuver to Improve Prediction of Conclusive Gastro‐Esophageal Reflux Disease
Source: Neurogastroenterol Motil. 2025 Jun 12;37(12):e70102. doi: 10.1111/nmo.70102 (PMC12623283; doi:10.1111/nmo.70102)

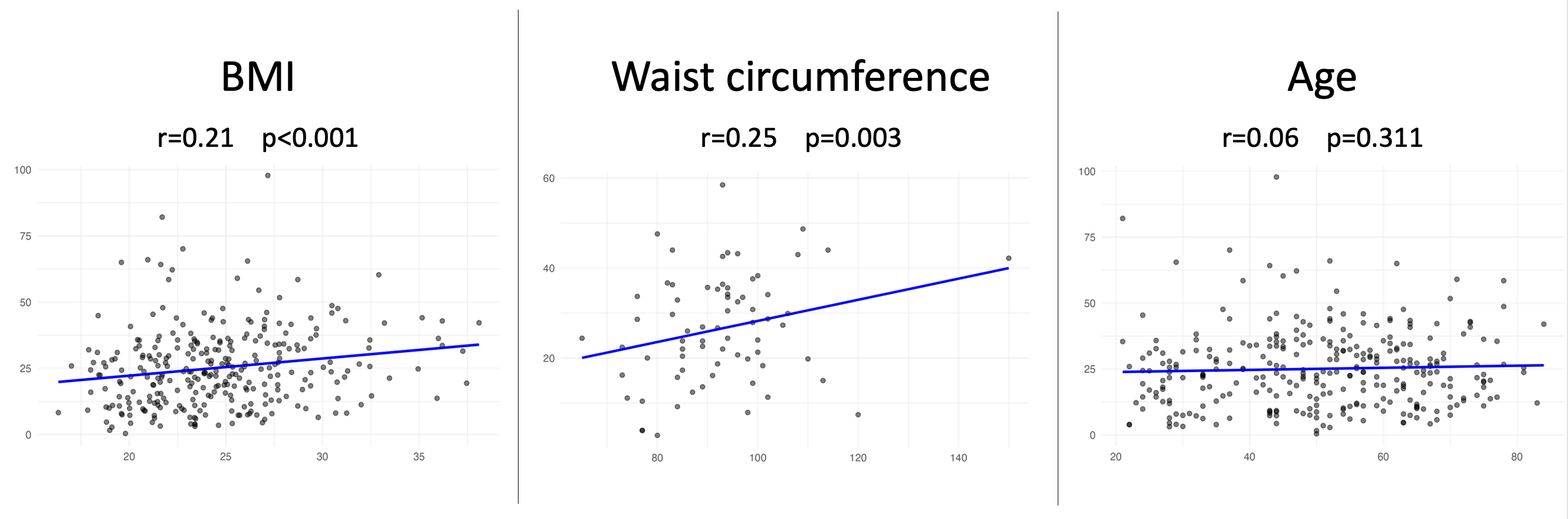

Supplement: Supplementary file 1 — Figure S1. Relationship between mean intra‐abdominal pressure increase and body mass index (BMI), waist circumference and age. [file NMO-37-e70102-s001.tiff]
